# Effect of Cold Plasma on Glial Cell Morphology Studied by Atomic Force Microscopy

**DOI:** 10.1371/journal.pone.0119111

**Published:** 2015-03-24

**Authors:** Nina Recek, Xiaoqian Cheng, Michael Keidar, Uros Cvelbar, Alenka Vesel, Miran Mozetic, Jonathan Sherman

**Affiliations:** 1 Department of Surface Engineering and Optoelectronics, Plasma laboratory, Institute Jozef Stefan, Ljubljana, Slovenia; 2 Jozef Stefan International Postgraduate School, Ljubljana, Slovenia; 3 Department of Mechanical and Aerospace Engineering, The George Washington University, Washington, D.C., United States of America; 4 Department of Neurosurgery, The George Washington University, Washington, D.C., United States of America; LAAS-CNRS, FRANCE

## Abstract

The atomic force microscope (AFM) is broadly used to study the morphology of cells. The morphological characteristics and differences of the cell membrane between normal human astrocytes and glial tumor cells are not well explored. Following treatment with cold atmospheric plasma, evaluation of the selective effect of plasma on cell viability of tumor cells is poorly understood and requires further evaluation. Using AFM we imaged morphology of glial cells before and after cold atmospheric plasma treatment. To look more closely at the effect of plasma on cell membrane, high resolution imaging was used. We report the differences between normal human astrocytes and human glioblastoma cells by considering the membrane surface details. Our data, obtained for the first time on these cells using atomic force microscopy, argue for an architectural feature on the cell membrane, i.e. brush layers, different in normal human astrocytes as compared to glioblastoma cells. The brush layer disappears from the cell membrane surface of normal E6/E7 cells and is maintained in the glioblastoma U87 cells after plasma treatment.

## Introduction

Plasma is an ionized gas that is typically generated in high-temperature laboratory conditions. Recent progress in atmospheric plasmas has led to the creation of cold plasmas with ion temperature close to room temperature [1,2]. Cold atmospheric plasma (CAP) has been extensively studied in the treatment of cancer, with the goal of maximizing tumor cell death and minimizing the therapy’s effect to healthy tissue [3,4]. The reactive ionized species, such as OH•, H_2_O_2_, N_2_^+^, NO and O_2_•^-^are the main components of the cold plasma jet that provides for therapeutic effects, not only with cancer, but also with biological disinfection [5], viral destruction [6] and wound healing [7]. It is well-known that NO is an omnipresent intercellular messenger in all vertebrates, modulating blood flow, thrombosis, neuronal activity, immune response, inflammation, and plays a critical role in tumorigenesis by modulating the apoptotic machinery [8–11]. According to Pacher and co-workers, NO and superoxide (O_2_^–^) can easily form peroxynitrite (ONOO^–^) once they collide or even locate within a few cell diameters of each other [12]. Peroxynitrite is a powerful oxidant and nitrating agent that is known to be a much more damaging to the cells than NO or superoxide, because cells readily remove superoxide and NO to reduce their harmful effects, while fail to neutralize peroxynitrite [13]. According to Lukes et al, the formation of NO2•, NO• and OH• radicals and NO+ ions by the discharge of plasma are at the gas-liquid interface and in the liquid [14]. Consequently, the generation of a moderate flux of peroxynitrite over long periods of time would result in substantial oxidation and potential destruction of host cellular components leading to a deregulation of critical cellular processes, disruption of cell signaling pathways, and induction of the cell death through both apoptosis and necrosis [15]. Nevertheless, there is still some controversy with respect to the mechanism of plasma—cell interaction. Some authors are of the opinion that ion species have the most important role in plasma–cell interactions by triggering intracellular biochemistry [16]. Alternatively, others have suggested that neutral species have the primary role in some plasma–cell interaction pathways [17]. Furthermore, the effects of various ion species may be highly selective; different species can have either ‘plasma-killing’ (such as O) or ‘plasma-healing’ (such as NO) effects [2,18]. The role of other species, such as O_3_ and OH, are not yet clear. Even less clear is the nature of the interaction between cold plasmas and cancer tissue. Only limited research into the utility of cold plasma for cancer therapy has been performed. For the most part, these in vitro studies are limited to skin cells and simple cellular responses to the cold plasma treatment [4,19,20]. In addition, preliminary reports on plasma’s in-vivo antitumour effect are reported [21]. Recent studies have delineated the effects of cold plasma on both the cellular and sub-cellular levels. On the cellular level, plasma effects include apoptosis, detachment of cells from the extracellular matrix and decreased migration velocity of cells. On the sub-cellular level, cell surface integrin expression is reduced [22,23], cell membrane permeability and consequent destruction is induced [16,24].

Glioblastoma, which is classified as grade IV astrocytoma by the WHO, is the most common and aggressive malignant primary brain tumor in humans, involving glial cells and accounting for 52% of all functional tissue brain tumor cases and 20% of all intracranial tumors. Despite advances in treatment options combining surgical resection, radiotherapy, and concomitant alkylating chemotherapy, the prognosis for glioblastoma patients still remains dismal with a median survival of 14.2 months [25]. The disproportionate malignancy of glioblastoma is due to its invasive growth pattern and high inter-and intratumoral genetic heterogeneity [26,27]. Due to the current limited treatment options and poor prognosis, glioblastoma garners much interest by researchers to develop novel treatments and effective selective cancer therapies.

AFM is a powerful, non-destructive technique that can be applied to the study of a variety of materials of biological significance and/or biological origin [28]. Because of the combination of high resolution and the ability to work under physiological conditions, it is a potentially powerful tool for biochemical and biological research avoiding complex sample preparation procedures and artifacts connected to them when comparing to other techniques such as scanning electron microscopy (SEM) and transmission electron microscopy (TEM) [29]. Very high resolution in the vertical z axis allows the possibility of analyzing cells at the nanoscale spatial resolution. In addition, three-dimensional (3D) reconstruction of the sample surface at molecular resolution is achievable [30–36]. Furthermore, AFM can be used to measure the mechanical properties of cell membranes. At present, no other microscopic techniques are able to provide directly both structural information of a biological sample and related functional information at such high spatial resolution. The balance between the two depends on the type of the cell sample, and on the image settings and the type of experiments that are performed. Using AFM to image living cells, important information on the architecture of membranes, organelles, and cytoskeletal structures can be directly gathered, without the use of potentially interfering fluorescent labels or probes. In studies of microbial or mammalian cells, AFM has been used to obtain high resolution images of the soft outer membrane and even of cytoskeletal structures underneath the cell membrane [28]. In this study, AFM has been used to image fixed normal human astrocytes (NHA) and glioblastoma cells before and after plasma treatment, in order to illustrate the selective effect of cold plasma on cell shape and morphology. High resolution images of cell membrane surface is provided to further understand and explain the possible morphological differences between cancerous and normal glial cells, as well as the interaction of cold plasma with cell membrane surface.

## Materials and Methods

### Cold atmospheric plasma configuration

The cold plasma device invented at the George Washington University has been described elsewhere [22]. In general, it has a configuration of central powered electrode of 1mm diameter and a grounded outer electrode wrapped around the outside of a 4.5 mm diameter quartz tube, powered by AC high voltage (2–10 kV, ~30 kHz). Industrial grade helium (from Airgas) was used as feeding gas. The flow rate was maintained at 4.7 l/min. The output voltage was set to 3.16 kV.

### Cell culturing

The human brain glioblastoma cell line, U87, was kindly provided by Dr. Ferid Murad’s lab from the Department of Biochemistry and Molecular Biology at the GWU [37]. The normal human astrocytes, E6/E7, were generously donated by Dr. Andrew Parsa at The University of California San Francisco [29]. Both cell lines were cultured in Dulbecco’s Modified Eagle Medium (Life Technologies) supplemented with 10% (v/v) fetal bovine serum (Atlantic Biologicals) and 1% (v/v) Penicillin and Streptomycin (Life Technologies). Cultures were maintained at 37 ◦C in a humidified incubator containing 5% (v/v) CO2. Cells were observed under a Nikon Eclipse TS100 inverted microscope.

### Cell viability assay

Cell viability was monitored using the MTT assay (Sigma-Aldrich, M2128), which is a colorimetric assay for measuring the activity of mitochondria and cellular dehydrogenase enzymes that reduce 3-[4, 5-dimethylthiazol-2-yl]-2, 5-dyphenyltetrazolium bromide, MTT, to its insoluble formazan, giving a purple hue. Cells were plated into 96-well flat-bottomed microplates in 100 ul medium per well. Confluence of each well was ensured to be at ~40%. Cells were then incubated for one day to ensure a proper cell adherence and stability. Cells were rinsed with phosphate buffered saline (PBS, Lonza 17–512F), replaced with a fresh medium before treated with cold plasma. After an additional incubation at 37 C for 24, 48, and 72 h post-plasma treatment, original culture medium was aspirated, 100 ul of MTT solution per well (7mg Thiazolyl Blue Tetrazolium Blue in 10ml medium for one plate) were added into each well. Reactions were maintained for 3 h at 37°C. The MTT solution were aspirated and 100 ul of MTT solvent (0.4% (v/v) HCl in anhydrous isopropanol) was added to each well to dissolve formazan crystals. Reactions were monitored by the Synergy H1 Hybrid Multi-Mode Microplate Reader at 570 nm. The entire set of experiments was repeated three times.

### Sample preparation and Atomic force microscopy (AFM)

AFM (Asylum, MFP-3D, California) was used to characterize the surface topography of the cell samples. AFM images were recorded in phosphate buffer solution (PBS) at room temperature. All measurements were done in contact mode using sharpened silicon nitride probes TR400PSA (Veeco—OTR4, Olympus, Japan) with two sets of two triangular levers. Resonance frequency of the shorter, 100 μm lever is 21–52 kHz and has a force (spring) constant of 0.02–0.23 N/m; for the longer, 200 μm lever the resonant frequency is 7–15 kHz and has a force constant of 0.01–0.05 N/m. The image data were collected in the deflection and topographic mode with the loading force of 1 to 5 nN at scan rate of, typically, 0.9 Hz. The images obtained in the deflection mode enhance structural details, whereas topographies show height of contours.

Cells were grown to near confluence on glass coverslips and subsequently imaged in 10mM PBS. After 48h of incubation in the culture medium (DMEM) supplemented with 10% (v/v) fetal bovine serum (Atlantic Biologicals) and 1% (v/v) Penicillin and Streptomycin (Life Technologies) at 37°C, 5% CO2, cells were attached to glass coverslips, ready to be observed in the AFM. Before imaging, cells were rinsed in PBS, fixed and dehydrated in order to simplify the imaging process. Cell fixation was accomplished by treating the coverslips with 0.5% solution of Glutaraldehyde for 5 min then washing with 10 mM phosphate buffered saline (PBS), pH7.4. After fixation, cells were dehydrated in the different % of ethanol solution according to standard protocol, described elsewhere.[38] Dehydration of cells was a key step in order to lower the average height of cells and make topography imaging possible. Samples were kept in PBS in an incubator for wet scanning or used right away for the AFM investigation. Fixed cells were imaged in the PBS.

### Statistical analysis

Results were plotted using a Microsoft Excel software (2011 for Mac) as mean ± standard deviation. Student t-test was used to check the statistical significance (*p<0.05, **p<0.01, ***p<0.001).

## Results

### MTT assay

Both NHA E6/E7 and glioblastoma U87 cells were treated for various durations from 5s to 60s. The results of the MTT assay showed that after 72 h, around 80% of U87 cells died at 30s and 60s of plasma treatment, while E6/E7 cells remained 90% and 60% cell viability at 30s and 60s plasma treatment at 72 h post-treatment time point. The p values for both cell lines after 60s treatment after 72 h showed statistically significant compared to control, while the p value for 30s plasma treated E6/E7 was not (S1 Fig.). Consequently, the threshold needed for U87 cells is determined as 30s.

### AFM imaging

**Fig 1:**
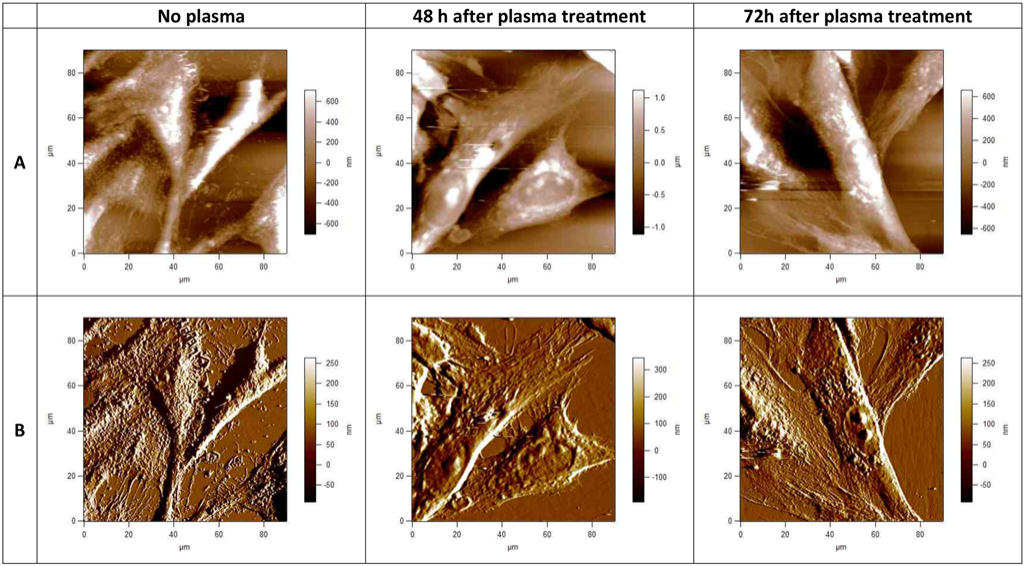
AFM images of fixed normal human astrocytes (E6/E7), taken using the Igor Pro 4, 90 x 90 μm scans. Upper row (A), topographies of 2-dimensional images of E6/E7 cells before and 48h and 72h after plasma treatment; lower row (B), corresponding deflection signal images. Cells were treated with cold plasma for 30 s. All images presented here were obtained in contact mode at room temperature and scanned in PBS.

**Fig 2:**
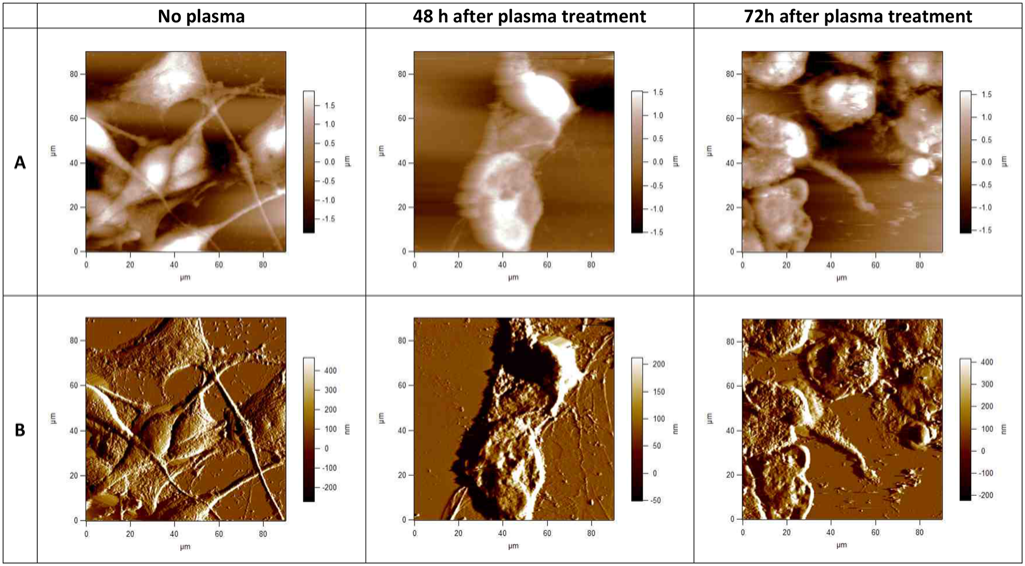
AFM images of fixed human brain glioblastoma (U87) cells, taken using Igor Pro 4 90 x 90 μm scans. Upper row (A), topographies of 2-dimensional images of U87 cells before and 48h and 72h after plasma treatment; lower row (B) corresponding deflection signal images. Cells were treated with cold plasma for 30 s. All images presented here were obtained in contact mode at room temperature and scanned in PBS.

Using AFM we observed morphological features on U87 and E6/E7 cells. AFM images were acquired in contact mode either as topographies that show the height of contours or as deflection signal images that highlight the fine features of surface morphology. A contact- mode AFM image of a mammalian cell, obtained under physiological conditions can yield several different images for the purpose of resolving the soft outer membrane of a cell from the cytoskeleton underneath. Here we present the height or raw topography image, and the corresponding ‘error’ or deflection signal image. Fig. 1A and Fig. 2A present topographical parameters of complete E6/E7 and U87 cells and Fig. 1B and Fig. 2B present corresponding deflection images. The information in the topography image (Fig. 1A and Fig. 2A) is dominated by the nucleus, although other features, such as the cell boundaries and the microvilli or invadopodia can be clearly distinguished. The information in the deflection signal image (Fig. 1B and Fig. 2B) shows the fine details of the cell structure. Untreated U87 and E6/E7 cells are extensively spread; cell body, shape, morphology, microvilli and invadopodia are clearly visible. The position of the cell bodies can be seen as well as where the cells meet (Fig. 1 and Fig. 2). Large fiber structures are seen on images of both U87 and E6/E7 adherent cells, although they are more pronounced on E6/E7 cells (Fig. 1). These structures are stress fibers, i.e., bundles of actin filaments. The underlying network of filaments can still be recognized on these images of both E6/E7 and U87 cells, but as a background of the other features. Control, plasma untreated U87 cancerous cells (Fig. 2) adhered to substrate created clusters with cell extension (invadopodia) projecting toward other cells. The size of invadopodia can be up to 100 μm long. The cytoplasm of untreated U87 cells seems smooth and rounded, with small protrusions (i.e. invadopodia) and ill-defined underlying structures, making the cytoskeleton hardly visible. On the contrary, the cytoplasm of untreated E6/E7 cells is rough and highlights the smaller, sharper features on the cell surface. In this case it is mainly the microvilli, rather than the cytoskeleton, that forms these smaller details (Fig. 1).

Besides the heterogeneity specific for cancerous cells which can have a sarcomatoid or epitheloid phenotype, cellular morphology of U87 cells varied greatly after plasma treatment. If we look closer at U87 cells 48h after plasma treatment, we observe big differences when compare to control untreated U87 cells (Fig. 2). Cells are both shrunken and rounded up with predominantly raised nuclear regions. The stress bundles of actin filaments are shown very clearly, particularly around the cell edges. The details of cell boundaries can also be seen in the AFM image, and some rounded features corresponding to organelles under the surface. As compared to the untreated U87 cells, the actin filaments do not dominate the images. Sharp protrusions can be seen covering the cell surface, which is not clear if they correspond to invadopodia or, what is more possible, to the damaged parts of the cell membrane and other cell components. The cell nuclei appear to remain intact. The cytoplasm of the cell seems very rough and dense, which is another consequence of plasma treatment. Even more noticeable changes like cellular shrinking and alterations of the cytoplasmic structure are observed on the U87 cells 72h after plasma treatment. The cytoplasm of treated cells appeared to have a rougher surface and showed clearly the loss of cytoskeleton fibres in the direction of the growth. A significant number of granular elevations and bigger clusters are seen on the U87 cells and sample surface after plasma treatment, since cells suffered from apoptosis. These clusters represent parts of the cell membrane (i.e. invadopodia) damaged upon plasma treatment. Cells treated with cold plasma show the multiple perforations at the boarders (which was not noticed in the untreated cells), loss of nuclear structures (i.e. nucleoli) and roughening of nuclear texture was in general more pronounced. It is observed that bundles of actin filaments quickly polymerize or depolymerize, rapidly adding or removing the components of the cytoskeleton to different locations within the cell. After longer incubation time (72 h after plasma treatment), observed effects are similar to those after 48h, although changes to cell shape, cytoplasmic and nuclear were even more drastic and pronounced.

In contrast, as shown on Fig. 1, we observe almost no differences on E6/E7 cell shape and morphology after plasma treatment and 48h or 72h of incubation time. Cells maintain the healthy and normal shape, and the extracellular matrix which forms the cell connections seem to be unchanged. Most of the cell are aligned parallel to the grooves and show the typical bipolar shape. However, there is one important difference when compared to control untreated E6/E7 cells. One can observe stark difference on the cell membrane surface of E6/E7 plasma treated cells. Cytoplasm of untreated cells is rough, there is a surface texture apparent from the membrane, with many sharp protrusions covering the cells and also the sample surface. These protrusions correspond to microvilli. These protrusions are not observed on the E6/E7 cell after plasma treatment and the cytoplasm of the E6/E7 cells is flat and smooth. However, these changes do not importantly influence the viability of normal E6/E7 cells.

**Fig 3:**
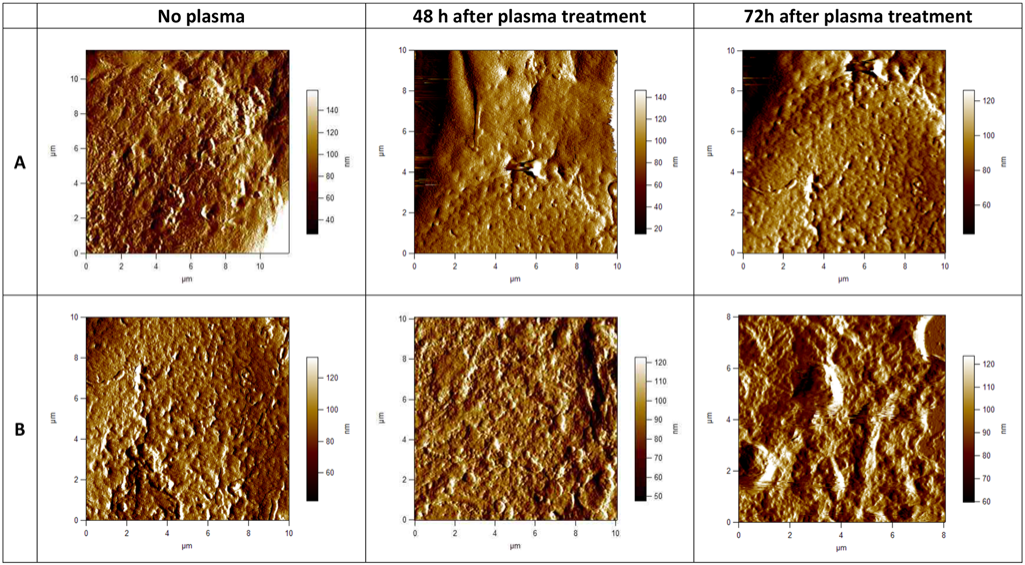
AFM images of cell membrane surface of fixed human astrocytes (E6/E7) and fixed human brain glioblastoma (U87) cells. Scan size is approximately 10x10 μm. In the upper row (A), 2-dimensional deflection signal images of cell membrane surface of E6/E7 cells are shown and in the lower row (B) 2-dimensional deflection signal images of cell membrane surface of U87 cells. All images presented here were obtained in the contact mode at room temperature and scanned in PBS.

To look closer on the cell membrane surface, AFM images of higher magnifications were performed. Smaller scans of a cell surface shown in Fig. 3 demonstrate more of the advantages of the AFM resolution. Fig. 3A and Fig. 3B shows high-magnification deflection images of E6/E7 and U87 cell membrane and cytoskeleton with scan size approximately 10x10 μm. If we compare cell membrane surface, stark differences can be observed in the cell membrane topography. Cell membrane surface of U87 untreated cells is flat, small pores in the membrane can be observed, no granular elevations. Looking closer on the image of U87 cells after plasma treatment, granular elevations can be observed, which are thought to be invadopodia and parts of the cell membrane and other cell components, damaged after plasma treatment and agglomerated in small clusters (Fig. 3B). On the contrary, we don’t observe any granular elevation on the surface of E6/E7 cells after plasma treatment. Pores on the cell membrane surface can be seen, without the presence of microvilli (Fig. 3A). According to the low magnification AFM images of E6/E7 cells (Fig. 1), granular elevations which are thought to be microvilli disappear from the cell surface after plasma treatment. However, there is more surface texture apparent from the membrane of untreated E6/E7 cells. Many sharp protrusions, i.e. microvilli, can be seen covering the cells (Fig. 1 and Fig. 3A).

**Fig 4:**
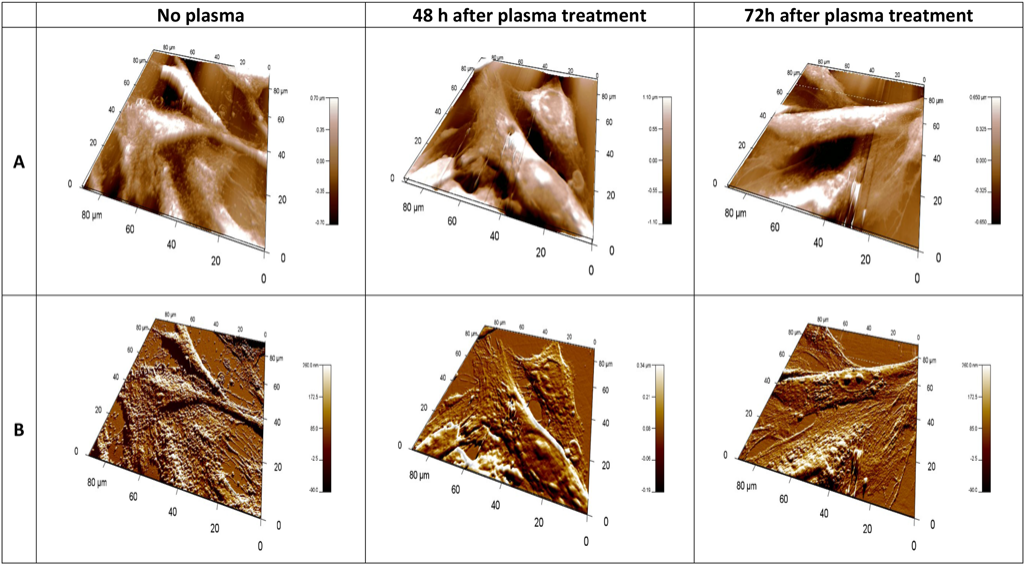
3-dimensional AFM images of fixed normal human astrocytes (E6/E7), taken using Igor Pro 4, 90 x 90 μm scans. In the upper row (A), topographies of 3-dimensional images of E6/E7 cells; lower row (B) corresponding deflection signal images. First column shows E6/E7 cells before plasma treatment, second column shows cells after 30s of plasma treatment, 48h of incubation time and third column after 30s of plasma treatment, 72h incubation time. All images presented here were obtained in contact mode at room temperature and scanned in PBS.

**Fig 5:**
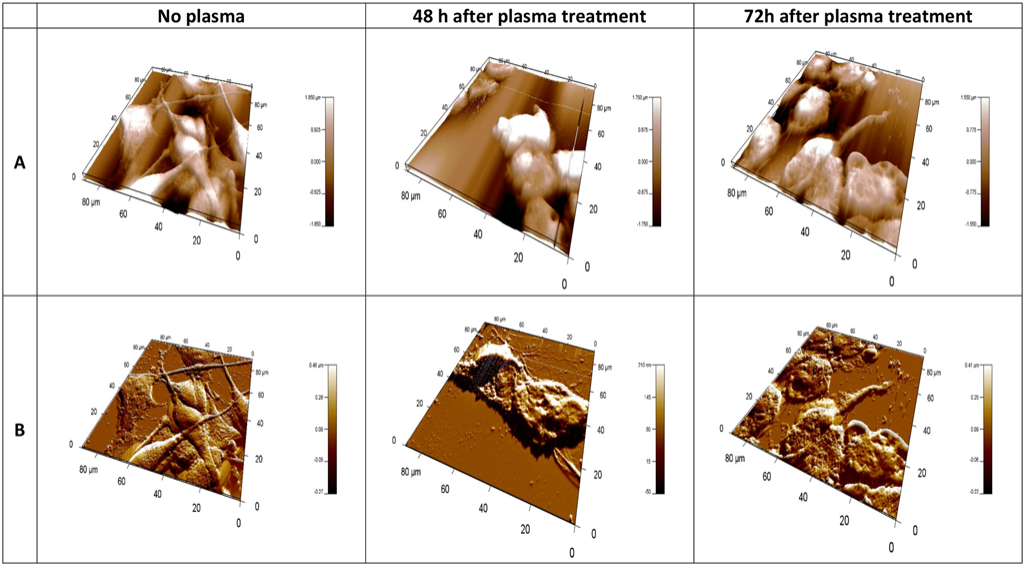
3-dimensional AFM images of fixed human brain glioblastoma (U87), taken using Igor Pro 4, 90 x 90 μm scans. In the upper row (A), topographies of 3-dimensional images of U87 cells; lower row (B) corresponding deflection signal images. First column shows U87 cells before plasma treatment, second column shows cells after 30s of plasma treatment, 48h of incubation time and third column after 30s of plasma treatment, 72h incubation time. All images presented here were obtained in contact mode at room temperature and scanned in PBS.

Using 3D topography images, complex information can be gleaned about superficial structure of cells. The height range over a cell is generally several microns, such that the three-dimensional topography image mainly shows the overall height of the cell. The altitudinal difference is about 1 μm for normal E6/E7 cells and 2 μm for U87 cells. This detail can be seen in Fig. 4 and Fig. 5. They represent 3D topographies (Fig. 4A and Fig. 5A) and deflection signal images (Fig. 4B and Fig. 5B) of E6/E7 and U87 cells before and after plasma treatment. 3D AFM images of E6/E7 cells on Fig. 4 correspond to the 2D images on Fig. 1. Similarly, 3D AFM images on Fig. 5 correspond to 2D images of U87 cells on Fig. 2.

## Discussion

This paper illustrates the interaction of the cold atmospheric plasma with the cell surface of NHA and glioblastoma cells and evaluates the effect of plasma on the cell shape and membrane as well as cell viability using AFM. Cold atmospheric plasma has been successfully shown to selectively induce apoptosis in cancerous cells, while leaving normal cells relatively unharmed [39]. The therapeutic effect is reported both with *in vitro* and *in vivo* experiments. Reactive oxygen species and reactive nitrogen species (ROS/RNS) were postulated to play a major mechanistic role in the CAP cancer therapy [40]. Previous research in CAP and cancerous cell interaction has repeatedly proven that there is a strong connection between CAP therapy and cell death [39,41]. In order to determine the threshold of plasma treatment on U87 cell death, normal human astrocytes (E6/E7) were used as the comparison cell line. Data from the previous research [42] showed, that the 30s plasma treatment caused 3-fold cell death in the U87 cells compared to the E6/E7 cells (S1 Fig.). For this reason the AFM images of U87 and E6/E7 cells presented in this paper were performed after 30s of CAP treatment.

AFM images of cells show a combination of surface and mechanical information. The AFM technique is particularly well suited to study the membrane surface, the cytoskeleton and dynamic reorganization of the cytoskeleton or cell surface structures [43]. The cell surface has been extensively imaged and 3D single vision of the cells topography in their natural environment and characterization of the membrane structures in situ with the good resolution are provided. Atomic force microscopy micrographs revealed important information on the shape and cell adhesion before and after plasma treatment. In order to assess differences in morphology, the morphological appearances of the adhered glioblastoma cells and NHA E6/E7 were imaged by AFM 48 and 72h after plasma treatment. AFM images of fixed human glioblastoma and NHA cells are obtained at 90x90 μm scans. Cells were grown to near confluence on glass coverslips and subsequently imaged in 10 mM phosphate buffered saline (PBS), pH 7.4. Once the cells are fixed, they are frozen in the state, and the cells can be imaged without changing state or functions. Fixation refers to the process by which proteins in the cell membrane and other cellular components are cross-linked with various reagents [44]. Fixation stops most or all biochemical processes within the cell, so these events cannot be studied with fixed cells. Unfortunately, the fixation process also kills the cell and changes its mechanical properties, rendering the outer membrane much stiffer, and making it less likely that structures such as cytoskeletal elements underneath the cell membrane can be detected [45]. Cells that have been fixed in such a manner are often used for SEM and AFM imaging because the fixation process strengthens the cell membrane dramatically and freezes it in a particular shape or morphology. Therefore, fixed cells can be imaged with the AFM more easily than live cells. Fixation by glutaraldehyde leads to a cellular death associated with an immobilization of the components and can modify the cell properties [44]. There are many different fixatives and protocols in use for fixation of biological material. Depend on the fixative and the concentrations of the fixative used, on the sample fixation time, different artifacts in morphology and in structure of biological samples can be observed. Based on the experimental results and observations from Moloney et al. [46], fixation method used was carefully chosen. In their experiment fibroblast cells in different fixatives were applied and morphological and structural changes were evaluated after fixation, together with artifacts observed. According to Moloney et al., Glutaraldehyde is the promising fixative, as there were no streaking, coating, and depression artifacts and saturation constraint observed. All these artifacts were absent, so the fibroblastic morphology and overall result were promising among the existing fixatives. With this fixation method, the AFM images are dominated much less by the underlying cytoskeleton and show the nucleus and more of the membrane surface details. Other membrane structures, such as ruffles, lamellipodias, invadopodia, microspikes and microvilli could be observed after cell fixation. Also sharp protrusions can be seen covering the cell surface (see Fig. 1 and Fig. 2). Minimization of the surface artifacts and thus maintenance of the surface features and morphology of the native sample was crucial point in the sample preparation. Consequently, substantial differences between the samples can more clearly be observed.

AFM images shows the specific impact of plasma on the cell shape and morphology after 48h and 72h of incubation time on U87 cells, which is significantly changed after treatment. On the other hand, NHA E6/E7 cells maintain their shape and morphology. We have observed important differences between surface textures, i.e. brush layers on the normal and cancerous cells. Even more interesting, this layer disappears from the surface of E6/E7 cells and is maintained in the U87 cells. These preliminary results suggest that differences in the architecture and assembly of cell membrane and cytoskeleton between NHA and glioblastoma cells are not only important for interactions with the environment, but probably play a crucial role in cell survival.

On the surface of NHA cells, smaller sharper features are observed. These features are microvilli, microscopic cellular membrane protrusions covering the plasma membrane. Present in normal cells, they increase the surface area of cells and are involved in a wide variety of functions including absorption, secretion, cellular adhesion and mechanotransduction [47]. Microvilli are formed as cell extensions from the plasma membrane surface and are covered with a glycocalyx, consisting of peripheral glycoproteins. This layer may be used to aide binding of substances needed for uptake, to adhere nutrients or as protection against harmful elements and external stress [48], e.g. plasma treatment in our case. The destruction of microvilli can occur in certain diseases because of the rearrangement of the cytoskeleton in host cells, which can lead to malabsorption of nutrients and persistent osmotic diarrhea [49]. As such, microvilli have a crucial role in many physiological processes. We have observed that after plasma treatment, as a consequence of cell membrane damage, the microvilli disappear from the cell membrane surface of NHA cells (Fig. 1). Nevertheless, these cells maintain their shape and morphology, what indicates that destruction of microvilli is not lethal for the NHA cells. Moreover, it is believed the microvilli can be reestablished by the cells, after the cell no longer suffers from the stress conditions.

Another type of brush layer is observed on the surface of U87 cells. These are specialized subcellular structures, invadopodia, which are responsible for cancer invasion and metastasis. Invadopodia, or invasive foot processes, are actin-rich protrusions that localize matrix-degrading activity to cell-substratum contact points and have a crucial role in cell signaling, proteolytic, adhesive, cytoskeletal, and membrane trafficking pathways [50]. Although invadopodia are similar to microvilli in many respects and share many common protein constituents, there are several key differences between these organelles. Unlike microvilli, which can be found in cells under normal physiological conditions, invadopodia are found only in pathological states, most commonly in invasive cancer cells. They are only few in number, approximately between 1 to 10 per cell, whilst microvilli numbers can range between 10 to several hundred depending upon the cell type. To this end, short-lived microvilli do not cause major degradation of the extracellular matix, whereas invadopodia, which have a longer lifetime, serve to enhance tumor metastasis by inducing basement membrane disruption through local matrix degradation [51].

Both microvilli and invadopodia are part of the underlying cytoskeleton, which is a structure underneath the cell membrane that lends it support. The cytoskeleton is the primary determinant to the overall shape or morphology of the cell and forms a rigid network that controls and supports both the cell shape and also the cell movement [52,53]. The dynamic balance between polymerisation and depolymerisation of the subunits of the different cytoskeletal fibres allows the cells to support tension or compression, and also to react quickly to change the shape of the cell, the means by which cell motility occurs. The polymerization and depolymerization of actin filaments are, therefore, important in many normal and disease processes, including metastasis, which is the spread of malignant tumor cells from one organ or tissue to another location [28].

Cells without an extracellular matrix are more susceptible to external stimuli and stress, such as plasma treatment; a potential reason for the susceptibility of U87 cells to plasma therapy. Another possible explanation addresses to functions of invadopodia in cancer cells. As described above, they have a crucial role in cell signaling and many other cellular pathways. Although they do not disappear from the cell membrane surface after plasma treatment, the morphology and the cell shape of U87 cells changed significantly after 48h and even more drastically after 72h of incubation (Fig. 2). U87 cell viability is significantly altered after plasma treatment with many granular elevations and bigger clusters seen on the cells surface. However, we believe that these clusters are parts of the cell membrane and other damaged cellular components and not microvilli. As already mentioned above, cancerous cells possess invadopodia which form cell extensions toward other cells to make the connections between each other. As seen from the AFM images, these connections are perturbed after plasma treatment. These results illustrate important morphological differences and membrane characteristics between NHA and glioblastoma cells and could be potential reason for resistance of NHA cells and sensitivity of U87 cells on plasma treatment.

These results indicate the potential role of CAP in the treatment of glioblastoma. Moreover, plasma does not influence the normal human astrocytes, which is of course beneficial and favorable for every cancer cell therapy. Furthermore, the AFM images validate the results of the MTT assay, which shows the selective apoptotic effect of plasma on the U87 cells.

The true mechanism of CAP on cancer cells remains to be elucidated. However, this study provides further data on the selective effect of cold plasma on glioblastoma cell viability by studying the cell surface details and morphology before and after plasma treatment with atomic force microscopy (AFM). Because of the extreme complexity in plasma-cell interaction, it is difficult to determine which mechanisms the cell uses to protect itself against stress (e.g. plasma treatment) and which signaling pathways are initiated inside the cell, but we have some indications what is happening on the cell surface, particularly with the cell membrane, after plasma treatment. This data will serve to better define the complex interaction between cold plasma and the cell membrane surface and aide in the design of future experiments. Imaging of other cell types using AFM looks to more clearly illustrate the effect of cold plasma on the cell membrane, cell morphology and cell viability.

## Conclusions

The fixed cell experiments presented here open new avenues for understanding the architecture and assembly of the cell membrane in normal human astrocytes and glioblatoma cells and contribute to understanding the complex interaction between plasma and cell membrane surface. These differences could be the possible reason for the selective effect of plasma on glioblastoma cells and may significantly contribute to cell viability after plasma treatment.

## Supporting Information

S1 Fig (a) 24, 48, 72 h MTT assay results of U87 treated with 5–60s duration of helium plasma jet (b) 24, 48, 72 h MTT assay results of E6/E7 treated with 5–60s duration of helium plasma jet (TIF)
