# Supplementary figures and images for: Effect of Cold Plasma on Glial Cell Morphology Studied by Atomic Force Microscopy
Source: PLoS One. 2015 Mar 24;10(3):e0119111. doi: 10.1371/journal.pone.0119111 (PMC4372419; doi:10.1371/journal.pone.0119111)

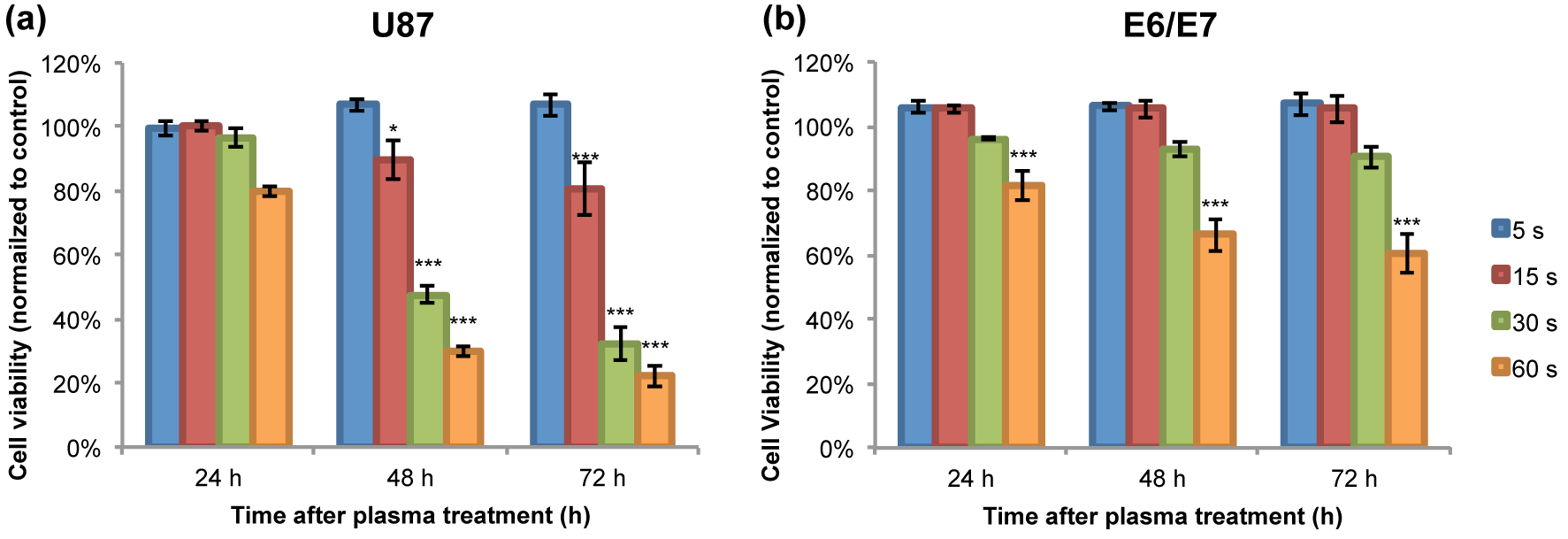

Supplement: S1 Fig — (TIF) [file pone.0119111.s001.tif]
